# Early diverging lineages within Cryptomycota and Chytridiomycota dominate the fungal communities in ice-covered lakes of the McMurdo Dry Valleys, Antarctica

**DOI:** 10.1038/s41598-017-15598-w

**Published:** 2017-11-10

**Authors:** Keilor Rojas-Jimenez, Christian Wurzbacher, Elizabeth Charlotte Bourne, Amy Chiuchiolo, John C. Priscu, Hans-Peter Grossart

**Affiliations:** 1 Department of Experimental Limnology, Leibniz-Institute of Freshwater Ecology and Inland Fisheries, Alte Fischerhuette 2, D-16775 Stechlin, Germany; 2 Universidad Latina de Costa Rica, Campus San Pedro, Apdo, 10138-1000 San Jose, Costa Rica; 3 Berlin Center for Genomics in Biodiversity Research, Königin-Luise-Straβe 6-8, D-14195 Berlin, Germany; 4 Leibniz-Institute of Freshwater Ecology and Inland Fisheries, Department of Ecosystem Research, Mϋggelseedamm 301 & 210, D-16775 Stechlin, Berlin, Germany; 5 Montana State University, Department of Land Resources and Environmental Sciences, 334 Leon Johnson Hall, MT 59717, Bozeman, USA; 6 Institute for Biochemistry and Biology, Potsdam University, Maulbeerallee 2, 14469 Potsdam, Germany

## Abstract

Antarctic ice-covered lakes are exceptional sites for studying the ecology of aquatic fungi under conditions of minimal human disturbance. In this study, we explored the diversity and community composition of fungi in five permanently covered lake basins located in the Taylor and Miers Valleys of Antarctica. Based on analysis of the 18S rRNA sequences, we showed that fungal taxa represented between 0.93% and 60.32% of the eukaryotic sequences. Cryptomycota and Chytridiomycota dominated the fungal communities in all lakes; however, members of Ascomycota, Basidiomycota, Zygomycota, and Blastocladiomycota were also present. Of the 1313 fungal OTUs identified, the two most abundant, belonging to LKM11 and Chytridiaceae, comprised 74% of the sequences. Significant differences in the community structure were determined among lakes, water depths, habitat features (i.e., brackish vs. freshwaters), and nucleic acids (DNA vs. RNA), suggesting niche differentiation. Network analysis suggested the existence of strong relationships among specific fungal phylotypes as well as between fungi and other eukaryotes. This study sheds light on the biology and ecology of basal fungi in aquatic systems. To our knowledge, this is the first report showing the predominance of early diverging lineages of fungi in pristine limnetic ecosystems, particularly of the enigmatic phylum Cryptomycota.

## Introduction

The diversity of fungi in freshwater, brackish, and marine environments has been suggested to be low in comparison to terrestrial habitats, with estimates of 3,000–4,000 species in total, composed mainly of members of Ascomycota, Chytridiomycota and to a lesser extent Basidiomycota^1–3^. However, there is increasing evidence that questions this limited fungal diversity in aquatic ecosystems. First, sampling efforts of fungi in aquatic habitats are low in comparison to terrestrial environments, and second, molecular analyses of environmental DNA samples using next-generation sequencing have revealed a large diversity of fungi whose sequences are not associated with known cultivated representatives in the reference databases^4^. In fact, an undetermined number of aquatic fungi are still missing in the taxonomy of the Fungal Kingdom, especially members of the early diverging lineages within Cryptomycota and Chytridiomycota^5,6^.

Aquatic fungi perform crucial roles in nutrient cycling, such as contributing to the flow of carbon through aquatic food webs and overall ecosystem functioning. Hence, their biodiversity and ecological role must be evaluated in detail for a comprehensive understanding of aquatic ecosystems^7,8^. Recent studies showed a high degree of fungal diversity in European freshwater lakes, where members of the basal phyla Chytridiomycota and Cryptomycota dominated the fungal community composition^9–11^. Similar results highlighting the importance of these groups have also been obtained in marine environments^12–15^. In particular, the early diverging fungal lineages have some physiological advantages for inhabiting aquatic ecosystems, including their mobility and capacity to parasitize numerous phytoplankton species such as diatoms, green algae, dinoflagellates and cyanobacteria^16,17^.

**Figure 1:**
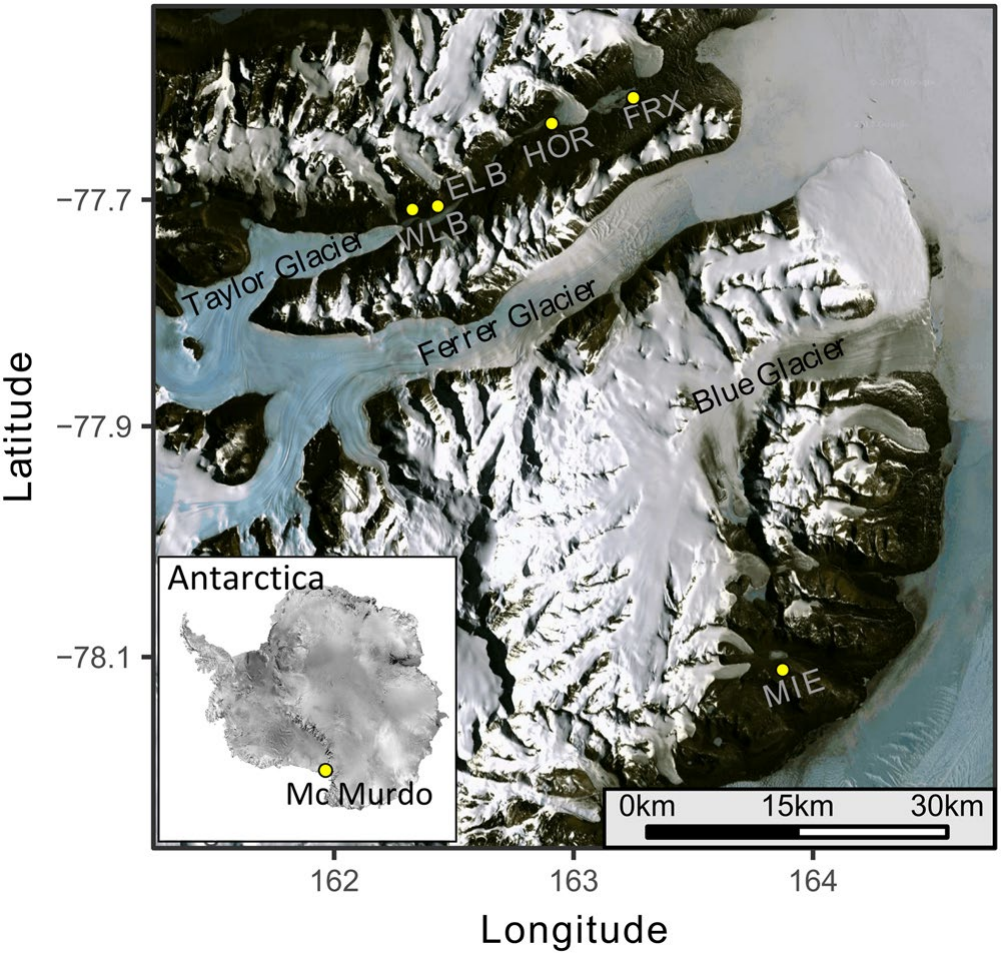
Geographical location of the five ice-covered lake basins studied in McMurdo Dry Valleys, Antarctica. This map was generated the R ggmap package^63^ importing satellite images from Google imagery©2017 NASA. ELB: Lake Bonney East lobe, WLB: Lake Bonney West lobe, HOR: Lake Hoare, FRX: Lake Fryxell, MIE: Lake Miers.

In this work, we studied the diversity and community composition of fungi in five perennial ice-covered lake basins of the McMurdo Dry Valleys, Southern Victoria Land, Antarctica. Particularly, we analyzed samples from Lake Miers, Lake Fryxell, Lake Hoare, and the West and East lobes of Lake Bonney (Fig. 1). These lakes not only constitute ideal models for studying the ongoing and future effects of global climate change on freshwater systems but also provide the unique opportunity to examine the ecology of aquatic fungal communities in extreme environments that have not experienced major alterations (including anthropogenic influence) for thousands of years.

The region of the McMurdo Dry Valleys is a polar desert with annual precipitation of 50 mm water equivalents and an average air temperature near −20 °C; this region constitutes the largest ice-free area in Antarctica with approximately 30% of the ground surface free of snow and ice^18,19^. The landscape is composed of glaciers, mountain ranges, ice-covered lakes, meltwater streams, arid patterned soils and permafrost, sand dunes, and interconnected watershed systems^20^. The landscape harbors numerous lakes with permanent ice caps of >3.5 m thickness that prevent wind mixing and significant nutrient inputs^21^.

The selected lakes form a hydrologically closed-basin system characterized by a strong and physically stable chemical stratification down the water column (meromictic) with oxygen-rich surface layers and sub-oxic and saline deep water^22,23^. The planktonic and benthic communities within these lakes are dominated by “microbial loop organisms” whose photosynthetic activity is a primary source of dissolved organic carbon. Previously, some studies examined the diversity of bacteria, archaea, protists, and ciliates, showing that communities are strongly stratified by depth^23–27^. In relation to fungal diversity, some works described fungal communities in surrounding soils^28–30^ or reported on the isolation of fungi using only cultivation-dependent methods^31,32^. Since only a minor fraction of the fungi in this aquatic ecosystem is cultivable, the community assembly and the role of fungi in these pristine lakes remain largely unknown.

In this work, we used Illumina sequencing of the 18S rRNA gene to characterize the fungal populations inhabiting five ice-covered lake basins in geographically isolated areas of the McMurdo Dry Valleys, Antarctica. We also explored their relationships with other detected eukaryotes as the effect of variables, such as lake type, depth, and habitat type (i.e., brackish vs. freshwater), on the community composition. We hypothesized that water column stratification would structure fungal community diversity and expected the fungal community structure to be composed of many undescribed taxa dominated by early diverging lineages of the Fungal Kingdom.

## Results

### Basic characterization of the lakes

**Figure 2:**
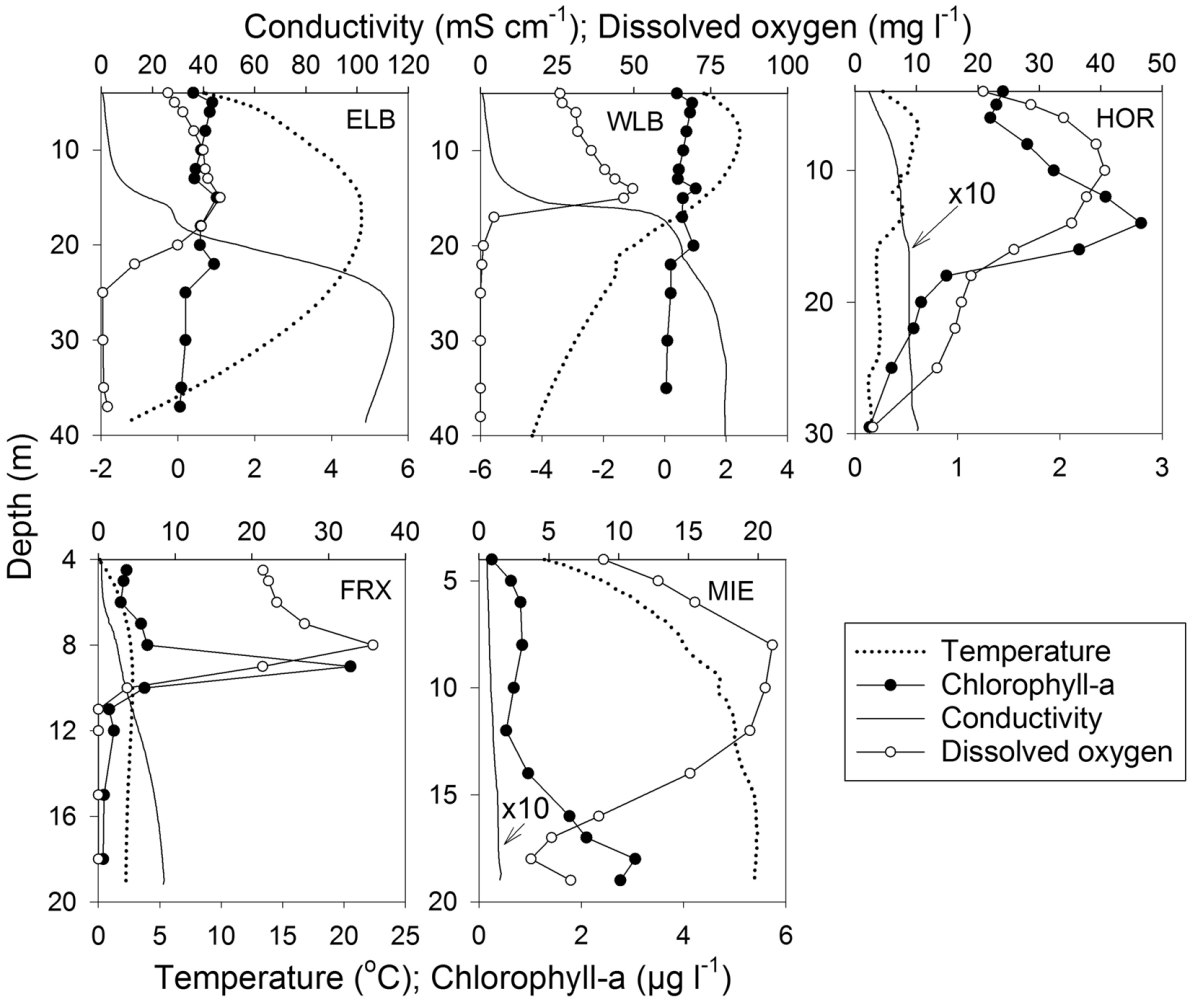
Profiles of conductivity, temperature, chlorophyll-a, and dissolved oxygen in the five lake basins studied during December 2011. All profiles begin at the ice-water interface. ELB: Lake Bonney East lobe, WLB: Lake Bonney West lobe, FRX: Lake Fryxell, HOR: Lake Hoare, MIE: Lake Miers. Note that the actual conductivity values presented for Lakes Hoare and Miers were multiplied by 10 to increase their visibility on the graph. Their actual values are 10 times lower than shown, revealing the freshwater nature of these two lakes relative to the others.

Vertical profiles of temperature, conductivity, chlorophyll and dissolved oxygen for the five chemically-stratified and permanently ice-covered basins studied are shown in Fig. 2. Lake Bonney has two distinct ~40 m deep basins separated by rather ~15 m deep sill that allows for mixing of freshwater between the trophogenic zones from each basin but restricts the exchange of the deep water brines^20^. The East lobe of Lake Bonney is characterized by fresh to brine waters that reach 114 mS cm^−1^ below the chemocline and water temperatures between 4.8 °C and −1.3 °C. The West lobe of Lake Bonney is influenced by the inflow of seasonal surface freshwater melt and cold (~−10 °C) subglacial brines from the Taylor Glacier^33^, producing a very sharp chemocline that reaches 80 mS cm^−1^ in the deeper layer and a vertical temperature range between 2.5 and −4.4 °C. Chloride is the dominant ion in Lake Bonney, leading to strong vertical stratification; pronounced variations also occurred for Br^+^, Ca^2+^, Cl^−^, Mg^2+^, Na^+^, and SO_4_^2−^ (Supplementary Figure S1). Lake Hoare (depth ~30 m) is relatively fresh (conductivity range 0.2 to 0.8 mS cm^−1^) with homogeneous temperatures (range 0. 6 to 0.2 °C). Lake Hoare is fed primarily by seasonal melt streams emanating from the Suess and Canada Glaciers^22^. Lake Fryxell (depth ~20 m) ranges from fresh surface waters to deep waters that reach 8.5 mS cm^−1^, whereby water temperatures range between 2.8 and 0.9 °C. This lake is fed by numerous glacially fed streams and has the highest volumetric phytoplankton biomass (20 µg Chl l^−1^) of all the lakes due to the inorganic nutrient and organic matter input from these streams together with the diffusive flux of these constituents from deeper waters^34,35^. The water below 10 m in Lake Fryxell is highly anoxic and contains sulfide levels in excess of 1.2 mM^36^. Lake Miers (depth ~19 m) is also a freshwater lake with maximum conductivities reaching 1.8 mS cm^−1^ near the bottom and relatively higher water temperatures (range 1.4 to 5.4 °C). This lake is located at a higher altitude in the Miers Valley, approximately 50 km south of Taylor Valley, and is fed by seasonal melt streams fed by the Miers and Adams Glaciers^37^. Lake Miers is the only lake in our study that has an outflow to the sea; all other lakes are hydrologically terminal and lose water only to ablation from the surface of the permanent ice cover. Biological production in Lake Bonney has been shown to be highly phosphorus deficient, whereas the other lakes are deficient in both nitrogen and phosphorus^35,38^.

### Proportion of fungi in relation to total eukaryotic community

**Figure 3:**
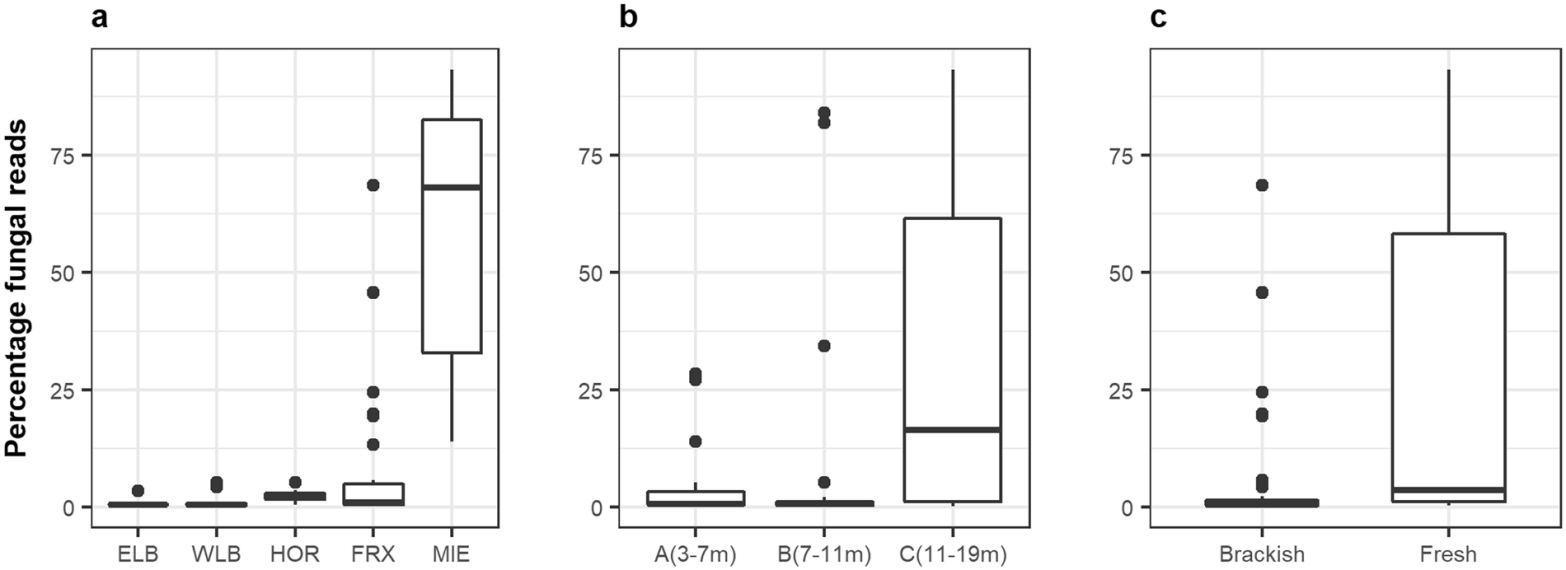
Percentage of sequences assigned to fungal taxa compared to the total number of eukaryotic sequences, estimated for each lake (**a**), depth layer (**b**) and aquatic habitat (**c**). ELB: Lake Bonney East lobe (n = 5), WLB: Lake Bonney West lobe (n = 19), FRX: Lake Fryxell (n = 27), HOR: Lake Hoare (n = 7), MIE: Lake Miers (n = 12).

From the 4.99 million 18S rRNA sequences analyzed, 787,937 were classified as fungi (Table 1). However, large differences in the proportion of fungi were observed between sites, ranging from 60.32% in Lake Miers to 0.93% in the West lobe of Lake Bonney (Fig. 3). According to Kruskal-Wallis tests, significant differences in the relative proportions of fungi were determined between lakes (*P* = 7.86 e-07), depth layers (*P* = 0.011), and habitats (freshwater versus brackish water, *P* = 0.0002). Concerning depth, deep waters that form the monimolimnia had a higher proportion of fungi than the overlying mixolimnia and chemocline regions. In relation to salinity, freshwater contained a significantly higher proportion of fungi than brackish waters. Conversely, we found no significant differences (Kruskal-Wallis, *P* = 0.20) between the size fractions (particle associated > 5.0 µm and free living: 0.2–5.0 µm), nor between the proportions of fungi obtained from DNA or RNA samples (Kruskal-Wallis, *P* = 0.34).

**Table 1:** Number of samples and proportion of fungal sequence reads relative to the total number of eukaryotic reads obtained from each lake basin.

Lake	Samples	Eukaryotes	Fungi	Percentage
ELB	5	364,214	3,645	1.00
WLB	19	1,081,761	7,385	0.93
FRX	27	2,085,178	161,495	8.05
HOR	7	434,806	9,552	2.40
MIE	12	1,022,850	605,860	60.32
**Total**	**70**	**4,988,809**	**787,937**	**14.01**

### Fungal community composition

**Figure 4:**
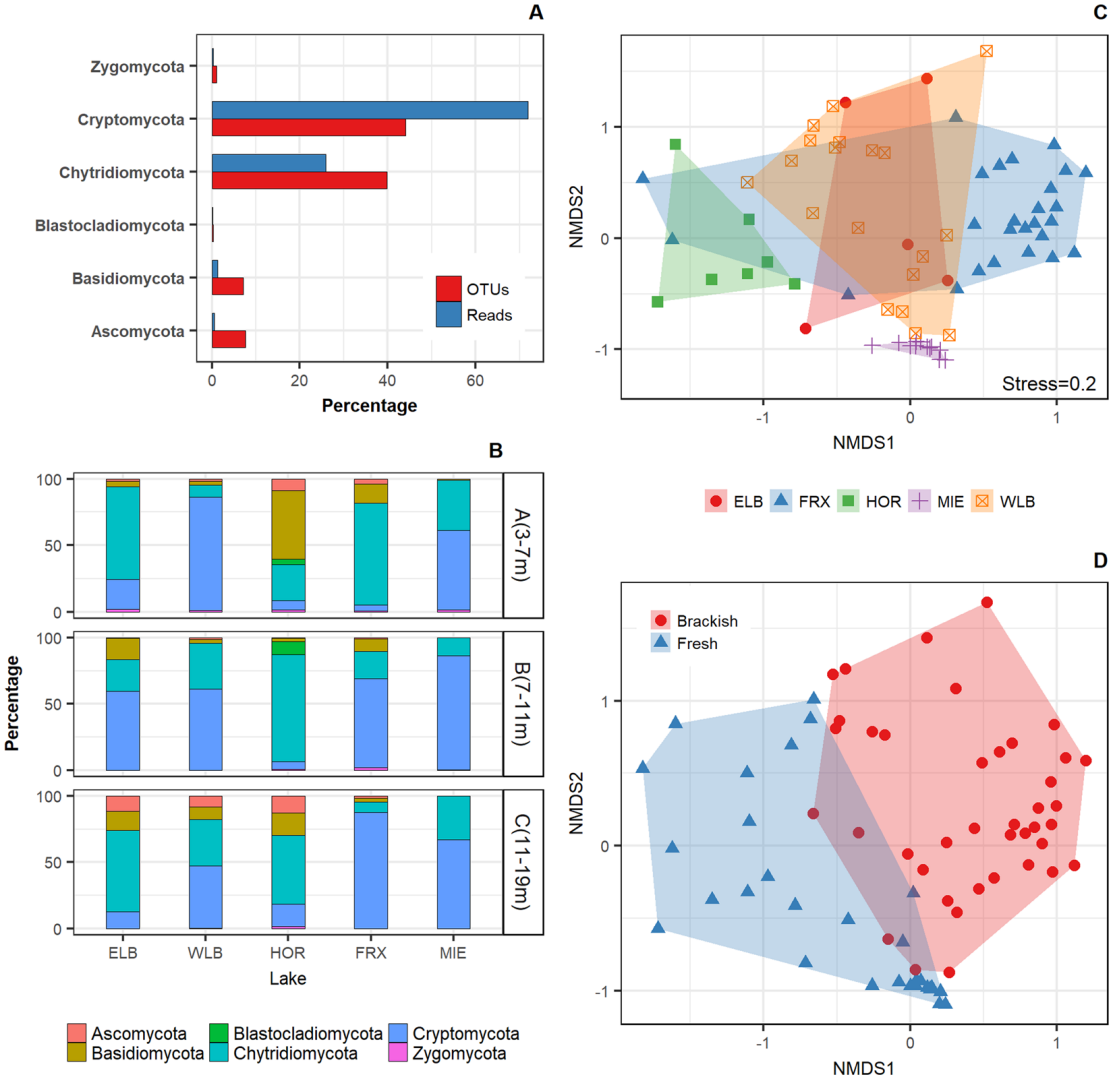
Fungal community composition in the Antarctic lake basins studied, considering the abundance distribution in terms of OTUs and sequence reads (**A**), composition by depth layer (**B**), NMDS multivariate clustering of communities according to the sites (**C**), and salinity of the habitat (**D**). ELB: Lake Bonney East lobe (n = 5), WLB: Lake Bonney West lobe (n = 19), FRX: Lake Fryxell (n = 27), HOR: Lake Hoare (n = 7), MIE: Lake Miers (n = 12).

Cryptomycota and Chytridiomycota were the most abundant fungal taxa in the McMurdo Dry Valley lakes. Cryptomycota represented ca. 72% of the fungal reads and ca. 44% of the fungal OTUs, while Chytridiomycota represented ca. 26% of the fungal reads and ca. 40% of the OTUs (Fig. 4). Two single OTUs, out of the 1313 identified at a distance cutoff of 0.03, comprised 74% of the fungal sequences: LKM11 Anta002 (Cryptomycota) comprised 52.6% and Chytridiaceae Anta004 (Chytridiomycota) comprised 21.4%. The remaining fraction was comprised of Ascomycota, Basidiomycota, Zygomycota, and Blastocladiomycota. In addition, we observed differences in the fungal community composition between and within lake basins. With different ratios, Cryptomycota and Chytridiomycota were the most abundant phyla. The phylum Cryptomycota was particularly dominant in lakes Miers, Bonney West and Fryxell, while the phylum Chytridiomycota was dominant in Bonney East and Hoare. The only exception to the dominance of basal fungi occurred in the upper layer of Hoare, where *Glaciozyma* sp. and *Mrakia* sp. (Basidiomycota) were more abundant, and extensive cyanobacterial mats were observed.

The analysis of the community composition showed that samples from the freshwater lakes Miers and Hoare formed individual clusters separated from the other lakes, while there was an overlap between the East and West lobes of Lake Bonney (Fig. 4). Most of the samples from Lake Fryxell were separated from the other groups, although some samples showed similarities to the Hoare’s cluster, as this lake presents a gradient of fresh surface waters to deep brackish waters. When analyzing the effect of salinity on the composition of the aquatic fungal communities, we observed a clear separation between the fungal populations in freshwaters and brackish waters. For example, we observed that members of Blastocladiomycota were found only in freshwaters. These results are consistent with the PERMANOVA tests, showing significant differences in fungal composition between lakes (*P* = 0.0009), depth layers (*P* = 0.0009), habitat (brackish vs. freshwater; *P* = 0.0009), DNA vs. RNA derived samples (*P* = 0.006), and between particle size fractions (*P* = 0.018). However, pairwise comparisons revealed no significant differences between the composition of the East and West lobes of Lake Bonney (*P* = 0.460) or between chemoclines and the deep layers (*P* = 0.094, additional pairwise analyses are provided in Supplementary Table S1).

### Richness and diversity

**Figure 5:**
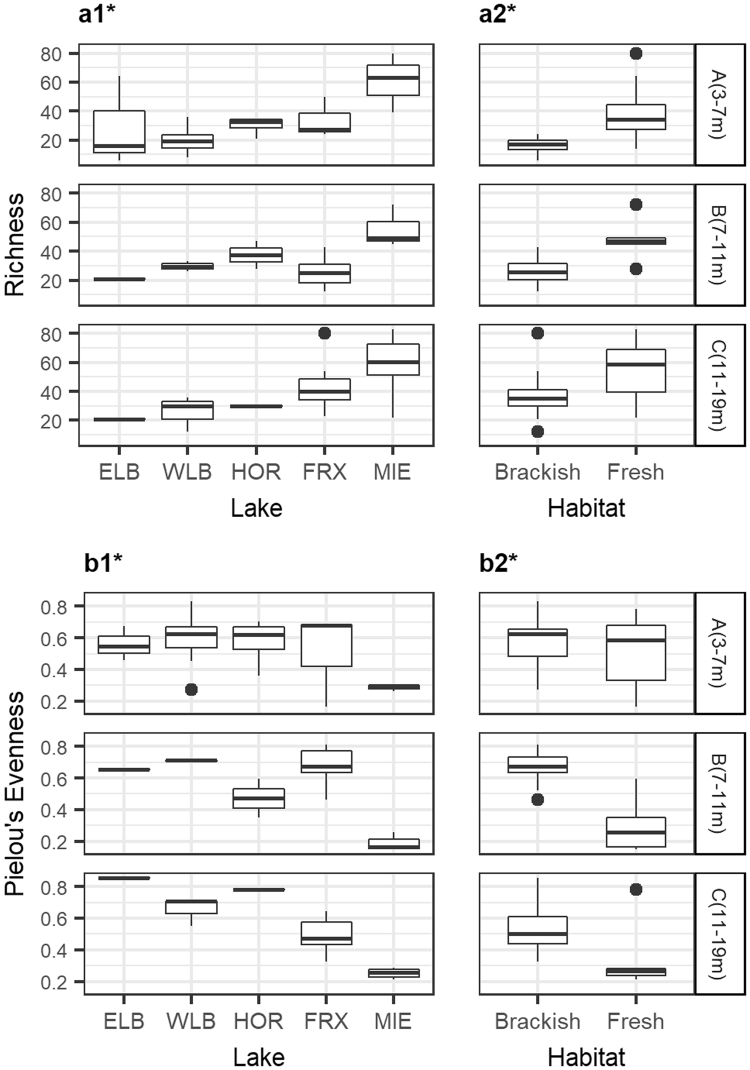
Distribution of the alpha diversity estimators according to depth layer. This includes richness per lake (**a1**), richness per habitat (**a2**), Pielou’s evenness per lake (**b1**) and Pielou’s evenness per habitat (**b2**). Asterisks indicate significant differences (Kuskal-Wallis Test, p < 0.05). ELB: Lake Bonney East lobe (n = 5), WLB: Lake Bonney West lobe (n = 19), FRX: Lake Fryxell (n = 27), HOR: Lake Hoare (n = 7), MIE: Lake Miers (n = 12).

We determined significant differences in fungal richness among the five lakes studied (Kruskal-Wallis, *P* = 2.301 e-05), with Lake Miers presenting the highest values (Fig. 5). However, pairwise comparisons showed no significant difference between the lakes located in the Taylor Valley (Dunn’s test adjusted with Benjamini-Hochberg method, *P* > 0.5), only between each lake and Miers. A similar situation occurred for estimates of the Shannon index and Pielou’s evenness. In relation to the habitat, Kruskal-Wallis tests showed that freshwaters presented a significantly higher species richness than brackish waters (*P* = 0.0001) but a lower evenness (*P* = 0.0016). Furthermore, we observed that with an increase in depth, there was a significant difference (*P* = 0.0079) between the richness values of both habitats.

### Network analysis

**Figure 6:**
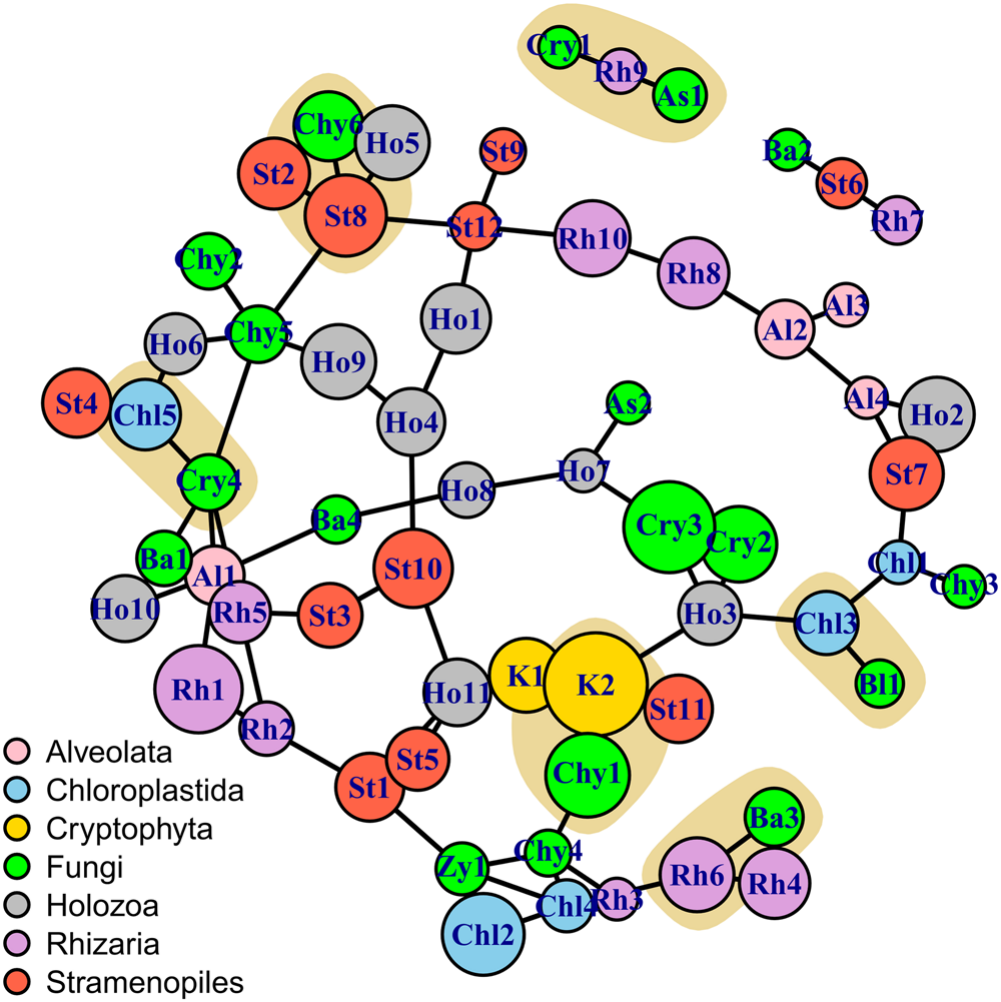
Network analysis highlighting the relationships within the fungal taxa and between fungi with respect to other eukaryotes cohabiting the lake basins. Colors of the nodes represent the taxa and the beige clouds highlight intergroup relationships, while the size of the circles is proportional to their log-abundance. Node names are composed of the initials of the taxonomic group and the consecutive number. We used SpiecEasi to generate the network of the 62 most abundant eukaryotic genera (comprising 98.2% sequences), which was then visualized with igraph. As:Ascomycota, Ba:Basidiomycota, Ba3:*Glaciozyma*, Bl:Blastocladiomycota, Chl2:Chlorophyta, Chl3:*Choricystis*, Chl4:*Paratrimastix*, Chl5:Prasinophytae, Chy:Chytridiomycota, Chy1:Chytridiaceae, Chy6:Rhizopidiales, Cry:Cryptomycota, Cry4:LKM15, K2:*Geminigera*, Rh6:*Heteromyta*, St8:*Parahysomonas*, Zy:Zygomycota, Zy1:Harpellales. The taxonomic description of the remaining nodes is shown in Supplementary Table S2.

We used network analysis to explore the relationships between the eukaryotic microorganisms co-habiting the lake basins (Fig. 6). This technique allowed us to visualize positive associations not only among members of the fungal taxa but also between fungi and other eukaryotes. For example, we identified strong relationships between chytrids (Chytridaceae) and *Geminigera* (Cryptophyta), the most abundant primary producer in this ecosystem. Other associations include chytrids (Rhizophydiales) and a heterotrophic stramenopile (*Parahysomonas*); LKM15 (Cryptomycota) and unicellular green algae (Prasinophytae); *Glaciozyma* (Basidiomycota) and the flagellate heterotroph *Heteromita* (Rhizaria); Blastocladiomycota and green algae *Choricystis*; Harpellales (Zygomycota), *Paratrimastix* (Chloropastida) and Chlorophyta. Because of this, and based on the nature of the organisms with which the fungi were interacting, we may infer that they can display roles as parasites, preys or saprophytic commensals. In addition, we detected that some fungi presented a high degree of connections with other organisms in the ecosystem, including *Hyaloraphidium*, LKM15, *Chytridium* and Harpellales.

## Discussion

This is the first report highlighting the predominance of Cryptomycota among fungal communities in an aquatic ecosystem. This predominance occurred in lake basins located in the Taylor and Miers Valleys of Antarctica, which are perennially ice-covered lakes presenting a minimum level of human disturbance and are characterized by strong physical and biochemical vertical gradients. The dominance of other early diverging lineages of fungi belonging to the phylum Chytridiomycota, has also been reported previously in communities of some specific freshwater and marine systems^9,12–15^. Hence, this finding led us to hypothesize that early diverging fungi might dominate fungal communities in the water column of undisturbed aquatic ecosystems, while members of Dikarya can more frequently dominate terrestrial habitats or aquatic environments affected by terrestrial input and anthropogenic activities.

Lake Miers contained the highest proportion of fungi, the highest species richness in all the depth layers, but the lowest values of evenness. This suggests that despite the many taxa inhabiting this lake, some species, mainly assigned to Cryptomycota, presented an unequal abundance distribution. The high richness in this lake could be explained by a combination of factors, including the freshwater habitat, the relatively warmer temperatures, and the location in a different valley with a higher altitude^37^. Other variables, such as light, have an effect on the high rates observed for thymidine uptake, which is considered a proxy of the phytoplankton primary productivity and might have also played a role, but this still requires further investigation^21,39^. We also observed an increased richness of fungi in the deeper layer of Lake Fryxell. In this case, despite the anoxic conditions, the higher phytoplankton biomass, DOC, and nutrient inputs from surrounding streams may have contributed to the higher values of richness and diversity observed^34,35^.

Aside from the lake ecosystem (site), the interaction of two factors shaped fungal richness and diversity in the McMurdo Dry Valley lakes: salinity and depth. Accordingly, higher values of richness are expected in freshwaters with respect to brackish waters or in the monimolimnion with respect to the overlying mixolimnion and chemocline regions. However, it is difficult to separate the specific effect of salinity from the effect of depth because these factors were closely related, since salinity varied with depth. A previous work also showed the effect of depth stratification on the bacterial communities of Lakes Fryxell and Bonney^25^. In addition, the effect of salinity on the mycoplankton was reported in other aquatic systems, showing higher diversity during periods of reduced salinity^12,40^. Salinity, however, might also provide additional niches where fungal groups can specialize and coexist^41,42^. In this regard, lakes Bonney and Fryxell provide ideal conditions to further investigate the mechanisms used by fungi to adapt to gradients of osmotic pressure (i.e., osmoregulation), high ion concentrations, and temperatures below 0 °C.

The fungal communities of the Antarctic ice-covered lakes studied were characterized by the dominance of a few highly abundant members of Cryptomycota and Chytridiomycota coexisting with a large number of low-represented organisms. In addition, we determined significant differences in the community composition among lakes, depth layers, and habitat. This suggests the development of certain niche preferences in some fungal taxa. The partitioning of the fungal communities according to different environmental parameters also has been observed in other temperate lakes^10^. The comparison of the fungal composition of the samples derived from RNA or DNA, obtained specifically from Lake Bonney, suggests that the fungal communities in this lake were effectively active, but they can vary with respect to the passive communities over time.

Network analysis showed a wide range of associations within not only fungal taxa but also between fungi and primary producers or as preys of other organisms. The most remarkable example was between a member of Chytridiacea, one of the most abundant fungal taxa, and *Geminigera*, the prominent cryptomonad primary producer in the lakes. In this case, we hypothesized that this is a parasitic relationship, since many members of Chytridiacea are obligate or facultative parasites^43,44^. Although co-occurrence does not necessarily imply a relationship, previous studies also showed Chytridiomycota as common phytoplankton parasites in both freshwater and marine ecosystems^5^. Furthermore, we were able to confirm a chytrid infection on algae by visualization with the fluorescence microscope (Supplementary Figure S2). Therefore, we estimate that parasitic fungi may play an important role in the regulation of phytoplankton biomass and the community structuring of the ecosystem, particularly considering the lack of phytoplankton grazers in these lakes.

According to our analysis, we detected some members of Rhizaria, Stramenopiles, and Holozoa that represent possible fungal predators. Some examples include *Paraphysomonas* and Rhizophydiales; *Spongomonas* and *Hyphozyma; Monogonta* and Saccharomycetales; and *Heteromita* and *Glaciozyma*. It is also possible that saprotrophic commensals, such as Harpellales, could use the byproducts of primary producers like *Paratrimastix* and Chlorophyta. Other examples include the linkages between the green algae *Choricystis* and Blastocladiomycota or between Prasinophytae and LKM15. In the case of LKM11, the other dominant fungus in the lakes, we observed no clear relationship with any primary producer, suggesting a saprophytic life style, as proposed for members of Cryptomycota^45^. Although these hypothetical relationships should be further examined, they show the versatility of fungi for using different carbon sources, but more importantly, they reveal the essential role of fungi as mediators in the transfer of energy and nutrients into the aquatic food webs^8,16,46^.

Finally, in this new era of increasing awareness of the importance of aquatic fungi in relation to their role in ecosystem functioning, this study provides new information on the community structure and the importance of the early diverging lineages of fungi in pristine ice-covered lakes of Antarctica. The composition of the fungal communities contained a higher proportion of Cryptomycota and Chytridiomycota, and this composition was influenced by site-specific effects, the stratification of the water column, and the habitat. Using network analysis, we are also able to extend current knowledge on the relationships of fungi with other eukaryotes and their relevant role in aquatic food webs. This study represents a contribution to better understand the dynamics of early diverging lineages of fungi in aquatic ecosystems. In addition, it emphasizes the need to examine with more attention the biology and ecology of these groups and to incorporate other independent but complimentary techniques such as cultivation, single cell isolation, microscopy, flow cytometry, and qPCR. Studies such as these will be particularly necessary to better characterize members of the Cryptomycota, for which until now only molecular information is available.

## Methods

### Sites and sample collection

The study was conducted on five lake basins in the Taylor and Miers Valleys (Fig. 1; Lake Bonney is comprised of two distinct basins) that are the focus of the McMurdo Dry Valleys Long-Term Ecological Research program (MCM LTER). During the austral summers of 2011–2012, we collected water samples (1–2 l) at selected depths through a ~30 cm diameter borehole in the ca. 4 m thick ice covers of each lake using sterile Niskin bottles. To prevent the introduction of contaminants into the lakes, we took precautions to drill just to the surface of the water. Prior to use, each corer was rinsed properly. For each lake a different sampler was used to avoid cross contamination. In addition, we established a suitable waiting period between the drilling and the water sampling. Then, the samples were filtered through 5.0 µm Puradisc Cellulose Nitrate syringe filters (Gelman Sciences, USA) and subsequently through 0.2 µm Sterivex filters (Millipore, USA) to distinguish between particle-associated and small free-living eukaryotes. Filters were stored with 2.0 ml of Puregene lysis buffer at −80 °C until further processing and nucleic acid extraction.

### Environmental data

All background environmental data and methods are available on the MCM LTER website (http://www.mcmlter.org/). A summary of the chemical and biological parameters used in this study are presented in supplementary information (Supplementary Figure S1). Briefly, temperature and conductivity were measured with a SBE25 Sealogger CTD as described previously^20^. Li^+^, Na^+^, K^+^, Mg^2+^, Ca^2+^, F^−^, Cl^−^, Br^+^, SiO_4_^4−^, SO_4_^2−^, and Fe^3+^ were measured by ion chromatography; dissolved oxygen was determined using the “azide modification” of the mini-Winkler titration; dissolved organic carbon (DOC) was measured on acidified samples with a Shimadzu TOC-V analyzer; phytoplankton primary production (PPR) was determined on light and dark bottles amended with ^14^C-bicarbonate and incubated *in situ* for 24 h^47^; bacterial production (TDR) was measured as ^3^H Thymidine and ^3^H Leucine incorporation^48,49^, and chlorophyll concentrations (CHL) were obtained with a submersible FluoroProbe (bbe-Moldaenke, Germany) spectrofluorometer.

### Nucleic acid extraction and sequencing

DNA from the microorganisms in the filters was extracted using a phenol-chloroform protocol^50^. From 12 samples of the West and East lobes of Lake Bonney, we also extracted RNA using an RNeasy Mini Kit (QIAGEN, Germany). The RNA was converted to cDNA with a One-Step RT-PCR Kit (QIAGEN, Germany) according to the manufacturer’s instructions. We amplified the V7 and V8 regions of the 18S rRNA gene using primers FF390 (5′-CGATAACGAACGAGACCT-3′) and FR1 (5′-AICCATTCAATCGGTAIT-3′), which showed good coverage of all fungal lineages^51^. For the 25 µl PCR reaction, we used a proof reading enzyme (Herculase II Fusion Polymerase, Agilent Technologies, Santa Clara, USA) and 40 ng DNA (or cDNA) as a template with the following conditions; 95 °C for 3 min initial denaturation followed by 35 cycles at 95 °C for 45 s, 52 °C for 1 min, 72 °C for 1 min, and a final extension at 72 °C for 5 min. The 96 resulting amplicons (~350 bp) went into the library preparation for Illumina MiSeq sequencing according to the protocol presented by the Illumina customer letter for 16 S sequencing with custom primers (Illumina guide to 16 S amplicon sequencing, Part # 15044223 Rev. A) and the Nextera index kit (Illumina, San Diego, USA). The samples were sequenced on a MiSeq sequencer (Illumina, San Diego, USA) with v3 2 × 300 nt chemistry. Sequences were demultiplexed with flexbar resulting in 6.4 M sequences. The sequence data were deposited into the NCBI Sequence Read Archive (BioProject 369175, BioSample accessions: SAMN06278024-SAMN06278119).

### Taxonomic identification and OTU generation

The 18S rRNA gene sequence reads were paired and quality filtered using Mothur 1.33.3 following the SOP tutorial for MiSeq sequencing data^52,53^. Subsequent processing included alignment of reads against the eukaryote subset (n = 16209) of the SILVA 123 data set^54^, pre-clustering (1 mismatch threshold), chimera removal with UCHIME^55^, and classified with SILVA 123, using the k-nearest neighbor algorithm with a minimum cutoff of 60%. We assigned sequences to OTUs using a split method based on taxonomy^56^. For this, sequences were clustered at the genus level in an initial step, and then all sequences within each taxa were assigned to OTUs according to the Vsearch method using a 0.03 distance cut-off^57^. After the first taxonomic classification obtained with the Mothur pipeline, we performed a second manual curation of the assignments. For this, a representative sequence of each OTU was further compared against the SINA Alignment and Classify service^58^. Notably, we found that many OTUs previously assigned as “Porifera”, “Unclassified Opisthokonta” or “Unclassified Nucletmycea” that are normally omitted in further analyses belonged to the Fungal Kingdom and contained large numbers of sequences. Other unclassified sequences at higher taxonomic levels were also compared against those in GenBank using the BLAST tool. The addition of this second step was fundamental for obtaining a proper and accurate taxonomic assignation of the eukaryotic organisms.

### Data analyses

Data processing, visualizations, and statistical analysis were performed in R^59^. To limit experimental biases due to sequencing depth, we removed samples whose total sequencing depth fell below the first quintile (1393 sequence reads), allowing us to keep an adequate number of sequences per sample but avoiding the loss of valuable samples of the lakes. This process resulted in the selection of 70 samples with nearly 4.99 million eukaryotic sequences. To estimate differences in the relative proportion of fungi and in the diversity indices, we used the non-parametric Kruskal-Wallis test, while pairwise comparisons were performed using Wilcox and Dunn’s tests (with Bonferroni adjustment). For further statistical tests, we normalized the data by converting the OTU counts (without singletons) into relative abundances. We used the Vegan package^60^ to perform non-metric multidimensional scaling (NMDS) analyses, permutational analysis of variance (PERMANOVA; functions *adonis* and *pairwise.adonis* with Benjamini-Hochberg adjustment), the Analysis of Multivariate Homogeneity of group dispersions and to calculate alpha diversity values. For the network analysis, we selected the 62 most abundant eukaryotic genera (17 classified as fungi), which corresponded to 98.2% of the total number of eukaryotic sequences. The analysis was performed with the SpiecEasi pipeline using the Meinshausen-Bühlmann neighborhood selection method^61^ and further visualized with igraph package using a Fruchterman-Reingold layout^62^.

## Electronic supplementary material

Supplementary Information
