# Supplementary material for: Early diverging lineages within Cryptomycota and Chytridiomycota dominate the fungal communities in ice-covered lakes of the McMurdo Dry Valleys, Antarctica
Source: Sci Rep. 2017 Nov 10;7:15348. doi: 10.1038/s41598-017-15598-w (PMC5681503; doi:10.1038/s41598-017-15598-w)
Supplement: Supplementary file 1 — Supplementary Information [file 41598_2017_15598_MOESM1_ESM.pdf]

Supplementary information

Early diverging lineages within Cryptomycota and Chytridiomycota dominate the fungal communities in ice-covered lakes of the McMurdo Dry Valleys, Antarctica

Keilor Rojas-Jimenez[1,2], Christian Wurzbacher[1,3], Elizabeth Charlotte Bourne[3,4], Amy Chiuchiolo[5], John C. Priscu[5], Hans-Peter Grossart[1,6*]

[1]Department of Experimental Limnology, Leibniz-Institute of Freshwater Ecology and Inland Fisheries, Alte Fischerhuette 2, D-16775 Stechlin, Germany

[2]Universidad Latina de Costa Rica, Campus San Pedro, Apdo. 10138-1000, San Jose, Costa Rica

[3]Berlin Center for Genomics in Biodiversity Research, Königin-Luise-Straβe 6-8, D-14195 Berlin, Germany

[4]Leibniz-Institute of Freshwater Ecology and Inland Fisheries, Department of Ecosystem Research, Müggelseedamm 301 & 210, Berlin,D-16775 Stechlin, Germany

[5]Montana State University, Department of Land Resources and Environmental Sciences, 334 Leon Johnson Hall, Bozeman, MT 59717, USA

[6]Institute for Biochemistry and Biology, Potsdam University, Maulbeerallee 2, 14469 Potsdam, Germany

***Author for correspondence**

Hans-Peter Grossart

Alte Fischerhuette 2, D-16775 Stechlin, Germany

Email: hgrossart@igb-berlin.de

Supplementary Figure S1

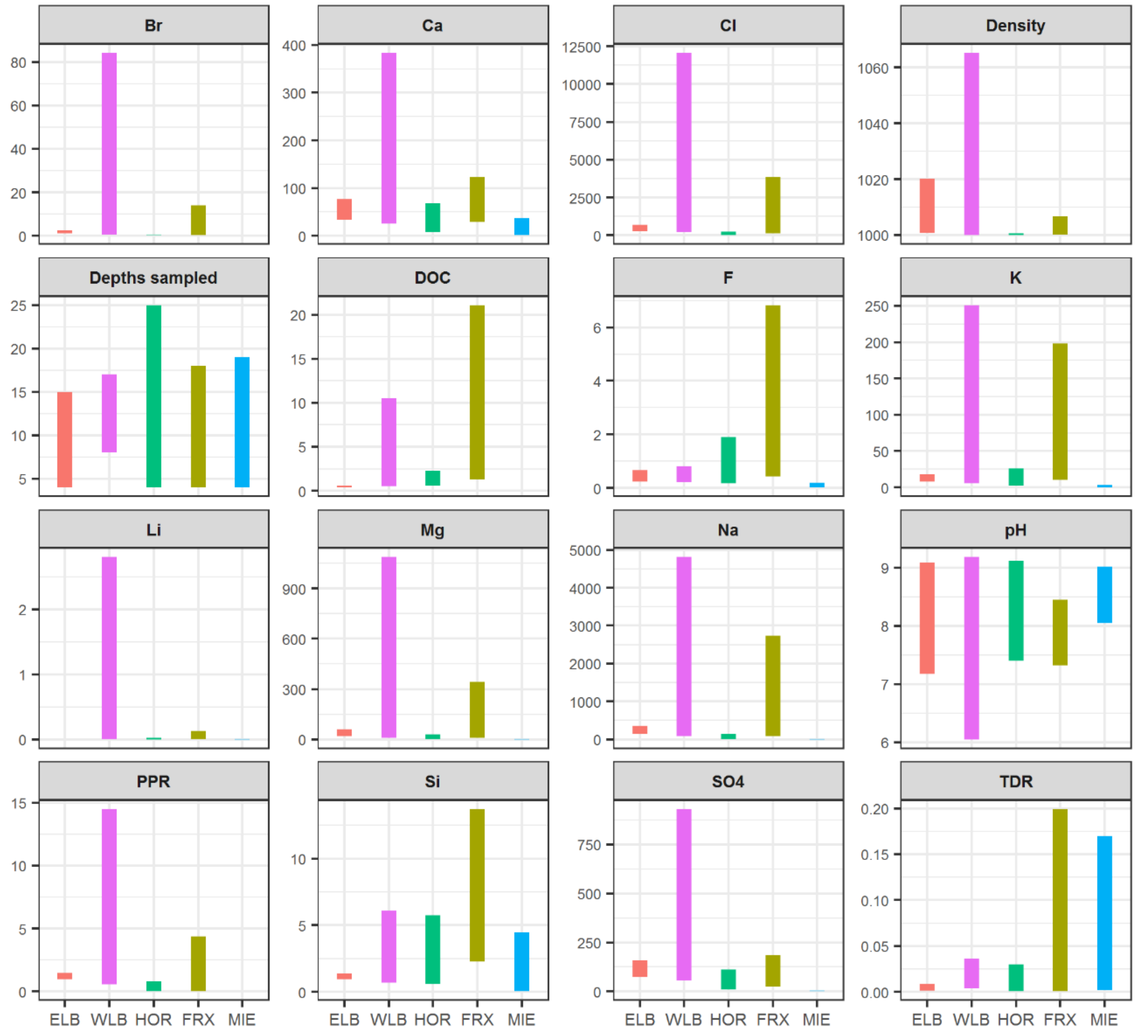

Supplementary Figure S1. Summary of additional environmental parameters measured in the five lake basins studied. Bars show the range of the values determined from all the samples of each lake. ELB: Lake Bonney East lobe, WLB: Lake Bonney West lobe, FRX: Lake Fryxell, HOR: Lake Hoare, MIE: Lake Miers. The units of DOC, Li, K, Mg, Ca, F, Cl, Br, Si, Na, and SO_4 are mg l^{-1}. PPR: phytoplankton primary productivity (µg C L^{-1} day^{-1}). TDR: thymidine uptake rate (nM TDR day^{-1}).

Supplementary Figure S2

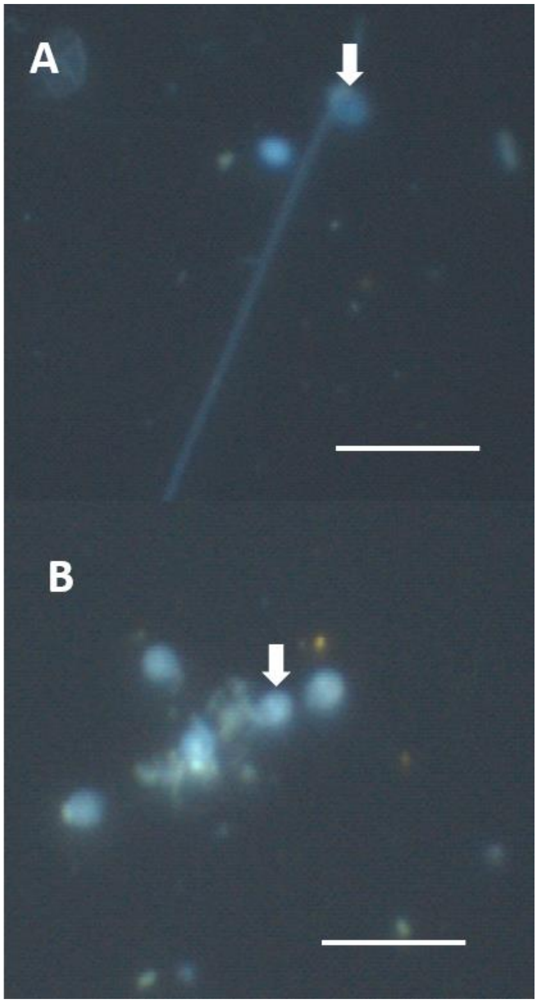

Supplementary Figure S2. Fluorescence microscope images of Chytrid-like fungi infecting algae in the East lobe of Lake Bonney (A), and in Lake Fryxell (B). Samples were stained with calcofluor white and observed under an epifluorescence microscope. Bars: 10 µm.

Supplementary Table S1

Supplementary Table S1. Statistical analysis of the fungal community composition related to different variables. The PERMANOVA tests were performed using functions adonis and pairwise.adonis (with Benjamini & Hochberg correction) implemented in Vegan package. Data were normalized by converting the OTU counts into relative abundances. Asterisks show significant differences.

**Variable**	**Pairs**	**F.Model**	**R2**	**p.value**	**p.adjusted**
**Lake***	FRX vs HOR*	10.17	0.25	0.001	0.002
	FRX vs WLB*	14.02	0.25	0.001	0.002
	FRX vs MIE*	25.55	0.42	0.001	0.002
	FRX vs ELB*	4.42	0.13	0.003	0.004
	HOR vs WLB*	6.01	0.20	0.001	0.002
	HOR vs MIE*	18.11	0.52	0.001	0.002
	HOR vs ELB*	2.65	0.21	0.006	0.007
	WLB vs MIE*	13.86	0.32	0.002	0.003
	WLB vs ELB	0.90	0.04	0.460	0.460
	MIE vs ELB*	7.93	0.35	0.001	0.002
**Depth***	A(3-7m) vs C(11-19m)*	7.10	0.13	0.001	0.001
	A(3-7m) vs B(7-11m)*	7.24	0.14	0.001	0.001
	C(11-19m) vs B(7-11m)	1.81	0.04	0.094	0.094
**Habitat***	Fresh vs Brackish*	10.39	0.13	0.001	0.001
**Filter***	5,0um vs 0,2um*	2.45	0.04	0.022	0.022
**Molecule***	RNA vs DNA*	3.20	0.13	0.011	0.011

Supplementary Table S2

Supplementary Table S2. Taxonomic description of the nodes used in the network analysis

Node	Taxonomy	Node	Taxonomy
Al1	Coccidia/Alveolata	Ho3	Calcarea/Holozoa
Al2	Cryptocaryon/Alveolata	Ho4	Choanomonada/Holozoa
Al3	Intramacronucleata/Alveolata	Ho5	Codosigidae/Holozoa
Al4	Vorticellides/Alveolata	Ho6	Craspedida/Holozoa
Chl1	Chlorophyceae/Chloroplastida	Ho7	Monogononta/Holozoa
Chl2	Chlorophyta/Chloroplastida	Ho8	Ploimida/Holozoa
Chl3	Choricystis/Chloroplastida	Ho9	Salpingoeca/Holozoa
Chl4	Paratrimastix/Chloroplastida	Ho10	Salpingoecidae/Holozoa
Chl5	Prasinophytae/Chloroplastida	Ho11	Stephanoecidae/Holozoa
Cry1	Paramicrosporidium/Cryptomycota	Rh1	Cercozoa/Rhizaria
K1	Cryptomonadales/Cryptophyta	Rh2	Cryothecomonas/Rhizaria
K2	Geminigera/Cryptophyta	Rh3	Glissomonadida/Rhizaria
Ba1	Agaricomycetes/Basidiomycota/Fungi	Rh4	Gymnophrys/Rhizaria
Bl1	Blastocladiales/Blastomycota/Fungi	Rh5	Hedriocystis/Rhizaria
Chy1	Chytridiaceae/Chytridiomycota/Fungi	Rh6	Heteromita/Rhizaria
Chy2	Chytridiales/Chytridiomycota/Fungi	Rh7	Protaspis/Rhizaria
Chy3	Chytridiomycetes/Chytridiomycota/Fungi	Rh8	Rhogostoma/Rhizaria
Chy4	Chytridium/Chytridiomycota/Fungi	Rh9	Spongomonas/Rhizaria
Ba2	Cryptococcus/Basidiomycota/Fungi	Rh10	Thecofilosea/Rhizaria
Cry2	Cryptomycota/Cryptomycota/Fungi	St1	Bicosoecida/Stramenopiles
Ba3	Glaciozyma/Basidiomycota/Fungi	St2	Bolidomonas/Stramenopiles
Zy1	Harpellales/Zygomycota/Fungi	St3	Chrysophyceae/Stramenopiles
Chy5	Hyaloraphidium/Chytridiomycota/Fungi	St4	Mallomonas/Stramenopiles
As1	Hyphozyma/Ascomycota/Fungi	St5	Nannochloropsis/Stramenopiles
Cry3	LKM11/Cryptomycota/Fungi	St6	Navicula/Stramenopiles
Cry4	LKM15/Cryptomycota/Fungi	St7	Ochromonas/Stramenopiles
Ba4	Mrakia/Basidiomycota/Fungi	St8	Paraphysomonas/Stramenopiles
Chy6	Rhizophydiales/Chytridiomycota/Fungi	St9	Pirsonia/Stramenopiles
As2	Saccharomycetales/Ascomycota/Fungi	St10	Spumella/Stramenopiles
Ho1	Acanthoecidae/Holozoa	St11	Stramenopile/Stramenopiles
Ho2	Adinetida/Holozoa	St12	Ulkenia/Stramenopiles
